# The Biomechanics of Glioblastoma: Why Glioblastoma Models and Clinical Reality Diverge

**DOI:** 10.3390/cells15100876

**Published:** 2026-05-12

**Authors:** Karina Köpke, Inge S. Zuhorn, Frank A. E. Kruyt

**Affiliations:** 1 European Research Institute for the Biology of Ageing (ERIBA), University Medical Center Groningen, Antonius Deusinglaan 1, 9713 AV Groningen, The Netherlands; k.kopke@umcg.nl; 2 Department of Biomaterials and Biomedical Technology, University Medical Center Groningen, Antonius Deusinglaan 1, 9713 AV Groningen, The Netherlands; 3 Department of Medical Oncology, University Medical Center Groningen, Hanzeplein 1, 9713 GZ Groningen, The Netherlands

**Keywords:** glioblastoma, biomechanics, extracellular matrix stiffness, preclinical models, magnetic resonance elastography, atomic force microscopy

## Abstract

**Highlights:**

**What are the main findings?**

- Glioblastoma stiffness measurements differ between preclinical and clinical studies, with preclinical approaches often reporting increased stiffness and clinical studies often reporting decreased stiffness.
- These differences are driven by both technical and biological factors.

**What are the implications of the main findings?**

- Better alignment of preclinical and clinical stiffness assessment is needed to improve the biological interpretation and translational relevance of GB mechanobiology.
- More physiologically relevant models, longitudinal measurements, and standardized experimental approaches are required to clarify the role of mechanical forces in glioblastoma progression and develop therapies that effectively target these mechanical factors.

**Abstract:**

Glioblastomas (GB) are highly aggressive brain tumors with poor patient prognosis and low survival rates. To identify novel therapeutic targets, the tumor microenvironment (TME) is increasingly examined, with a particular focus on biomechanical changes in the extracellular matrix (ECM) that contribute to GB aggressiveness. In GB, the ECM stiffens, regulating cell behavior through mechanotransduction. Preclinical in vitro and ex vivo studies generally report increased stiffness in GB relative to healthy brain tissue, whereas clinical in vivo measurements often report decreased stiffness. This review examines potential causes for this discrepancy, highlighting both biological and technical factors. Preclinical measurements are frequently performed using atomic force microscopy (AFM), while clinical stiffness is assessed via magnetic resonance elastography (MRE). Differences in methodology, including sample preparation, measurement modalities, and spatial scale, partly explain divergent stiffness values. Biological factors such as necrosis, edema, and physical confinement by the skull, which are preserved only in vivo, also contribute to these differences. To reconcile these findings, future research should employ physiologically relevant in vitro models that better replicate in vivo GB biomechanics, together with high-throughput and accurate animal models. Integrating these approaches may clarify the biomechanical landscape of GB and result in more effective therapeutic strategies.

## 1. Introduction

Glioblastomas (GB) are highly invasive brain cancers, for which treatment only offers modest improvements in outcome. GB has an annual incidence of approximately 3–4 cases per 100,000 person-years, with variation across regions and study designs. Globally, brain and central nervous system cancers accounted for an estimated 8.9 million disability-adjusted life years in 2021, highlighting their substantial health burden [1,2]. Chemo-radioresistance and cell dissemination lead to tumor recurrences within 2–3 cm of the original lesion margin, posing a challenge to finding effective treatment options. Interactions of GB cells with the tumor microenvironment (TME) play a pivotal role in GB cell spreading and regulatory mechanisms could serve as a potential therapeutic target [3,4]. Biomechanical properties of the tumor and its microenvironment, such as extracellular matrix (ECM) stiffening, have been associated with increased tumor aggressiveness and shorter patient survival [5,6,7,8]. ECM stiffening is a result of altered levels and composition of ECM components that dynamically regulate GB cell migration and proliferation through mechanosensitive signaling pathways [9,10,11,12], highlighting the importance of understanding how biomechanical changes drive invasion and potentially impact therapy response in GB.

Compared to other cancers, GB develops in a uniquely soft biomechanical environment because the brain is one of the softest tissues in the body, typically with stiffness values around 1–2 kPa [13,14]. Hence, even small stiffness changes create a significant mechanical contrast, influencing GB cell migration and invasion through mechanosensitive signaling [12]. This softness complicates both relevant disease modeling and accurate stiffness measurement. Preclinical culture systems are often stiffer than the native brain, potentially skewing cell behavior, while stiffness measurements of patient tumors in a clinical setting must be sensitive enough to detect subtle differences in a soft environment.

Interestingly, despite the brain’s intrinsic softness, many preclinical models and even ex vivo stiffness measurements of resected GB tissue report relatively high stiffness values. In contrast, in vivo techniques such as magnetic resonance elastography (MRE) frequently find GB tumors to be as soft as, or softer than, surrounding brain tissue. This apparent discrepancy poses substantial implications for basic research as well as clinical translation: if preclinical models do not accurately represent mechanical GB properties, conclusions about mechanobiology, drug response, and invasion dynamics may not translate to clinical reality.

This review briefly introduces the current understanding of ECM stiffening on GB aggressiveness, followed by describing the in vitro, in vivo and ex vivo models used to study this process, and the different methodologies to assess preclinical and clinical GB stiffness. Potential causes of observed discrepancies in stiffness measurements using different models and/or methods and the opportunities to guide more accurate model design are discussed in order to improve translational relevance.

## 2. Mechanistic Basis of Glioblastoma Stiffness

### 2.1. Molecular and Cellular Drivers of Glioblastoma Stiffness

Stiffness is a mechanical property defined as the resistance to deformation upon external force application [15]. In biology, stiffness can be assessed on a cellular as well as a tissue level. Cellular stiffness arises primarily from the mechanical properties of the cytoskeleton and the nucleus, whereas tissue stiffness includes the total stiffness of cells, their surrounding ECM, and possibly the surrounding fluids [16,17]. Increased tissue stiffness is a stressor and effector of cancer progression, e.g., through stimulating mechanisms that control cell proliferation, spreading and metastasis (reviewed elsewhere [18]).

**Figure 1:**
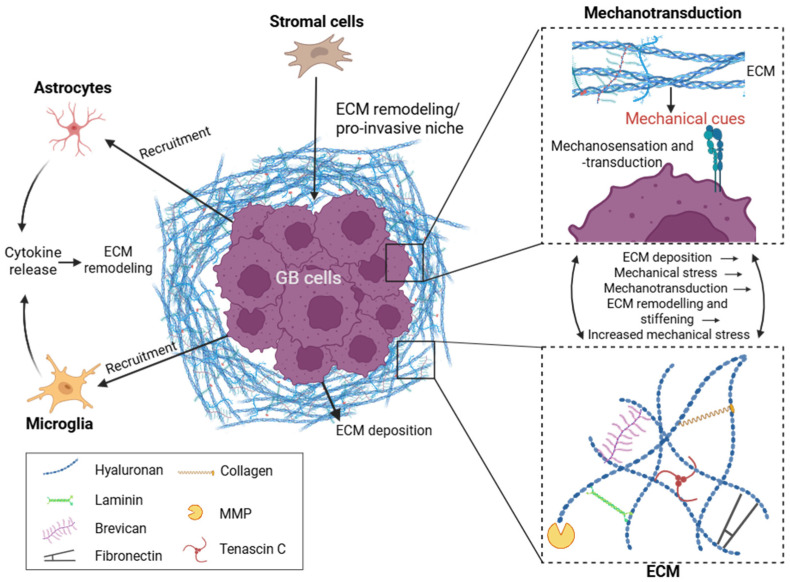
Glioblastoma (GB) tumor microenvironment (TME) interactions influencing the extracellular matrix (ECM). Schematic of GB cells embedded within a dense ECM network. Surrounding stromal and immune cells dynamically interact with tumor cells and contribute to ECM remodeling. ECM deposition by GB cells increases stiffness and mechanical cues, which activates mechanotransduction. Mechanotransduction in turn induces ECM remodeling via matrix metalloproteinases (MMPs), altering ECM structure and contributing to increases in ECM stiffness, which induces more mechanical stress. These reciprocal processes create a feedback loop in which ECM remodeling and cellular activity continuously shape each other. Modified mechanical properties influence GB cell migration, invasion, and signaling.

Stiffness in GB arises from reciprocal interactions between tumor cells, immune cells and stromal cell populations that remodel the surrounding ECM (Figure 1). Tumor cells contribute to stiffening through ECM deposition and matrix reorganization [10,19]. Immune cells, specifically microglia and macrophages, further amplify this process through cytokine release, supporting microenvironmental reorganization [20,21]. Further, stromal-like cells including mesenchymal-like stem cells and fibroblast-like populations actively contribute to matrix remodeling and the creation of a pro-invasive mechanical niche [22,23,24]. Changes in stiffness are thought to not only be a byproduct of GB tumor growth, but an active and dynamic driver of GB invasion by providing structural infiltrative pathways, in turn further influencing aggressive cellular behavior.

### 2.2. ECM Remodeling in GB

In the healthy brain, the ECM is flexible and serves mostly as a structural scaffold that is, amongst other things, involved in the regulation of cell differentiation and tissue homeostasis. In recent years, studies have highlighted the importance of ECM stiffness in GB progression [24,25]. During GB tumorigenesis and progression, the ECM undergoes significant compositional and mechanical remodeling, resulting in a mechanically more rigid microenvironment that facilitates GB cell growth and invasion [25,26]. Healthy brain ECM consists mostly of polysaccharides, proteoglycans and fibrous protein. Most abundantly present are Collagen, Hyaluronan (HA), as well as Tenascins and proteoglycans such as Aggrecan and Brevican. The expression and therefore abundance of these components are altered in GB, influencing the mechanical conditions of the tumor TME [10,25,27].

HA canonically provides integrity and structure in the brain ECM. During GB progression, HA expression is gradually increasing, as is the expression of HA receptor CD44 [28,29]. Increases in HA expression led to a denser and stiffer ECM, while higher HA production has been linked to increases in proliferation rates and cell motility [30,31,32,33]. Another important ECM component is tenascin C (TNC) which is highly expressed in GB compared to the normal human brain [34,35], leading to ECM stiffness modifications [35,36,37]. Its expression increases with tumor progression and grade and has been associated with high cell migration rates and invasion [30,32]. Inhibiting or decreasing TNC led to decreases in GB cell migration in vitro and in vivo [35,38]. Fibronectin (FN1) provides structural support in healthy brain ECM and is more abundantly present in GB when compared to the healthy adult brain [6]. Increased FN1 expression in GB has been linked to reduced overall survival in patients [39,40]. Deposition of FN1 by GB cells alters the mechanical properties of the ECM, enhancing cell adhesion and thereby promoting invasive behavior [10,41,42]. Furthermore, depleting FN1 in an orthotopic GB model reduced tumor growth [42]. Lastly, Matrix metalloproteinases (MMPs) such as MMP9 are overexpressed in GB and contribute to ECM remodeling through degradation of ECM components like collagens and laminins [43,44]. MMP-mediated breakdown of the ECM creates migration pathways that allow for tumor cell invasion [45,46]. Further, ECM degradation triggers new deposition of ECM components and crosslinking, which in turn increases ECM stiffness [47,48]. This overall process results in a positive feedback loop, as a stiffer ECM again leads to upregulation of MMPs [10,49].

Together, alterations in ECM components illustrate how dynamic expression changes drive ECM remodeling and stiffness in GB, prompting the question of the underlying mechanism by which tumor cells detect and respond to (mechanical) changes in their microenvironment.

### 2.3. Mechanotransduction Pathways in GB Cells

Mechanotransduction is the process by which cells sense mechanical cues and convert them into biochemical signals, enabling them to respond to microenvironmental properties such as stiffness, tension, and compression [50,51]. These cues are primarily sensed at the cell membrane through ECM-cell interactions involving integrins, CD44, and focal adhesions, which transduce the signal to the cytoplasm and ultimately to the nucleus through activation of mechanosensitive pathways including FAK/Akt, RhoA/ROCK and YAP/TAZ. Nuclear shuttling of YAP/TAZ, for example, regulates the expression of ECM-related genes such as TNC through chromatin remodeling and transcription factor activation, which in turn promotes ECM protein deposition, ultimately driving ECM remodeling [11,51,52]. In GB, ECM remodeling alters cytoskeletal tension and ECM rigidity which enhances mechanotransduction signaling. Continued mechanical stresses further drive adaptive cytoskeletal remodeling and motility, establishing a positive feedback loop in which ECM stiffening activates mechanotransduction pathways, thereby sustaining GB cell invasiveness [10,11,53,54]. Additionally, GB durotaxis is influenced by mechanical cues and subsequent mechanotransduction, resulting in stiffness-dependent cell morphology, migration speed and invasive behavior [55,56].

## 3. Experimental Models for Studying GB Stiffness

### 3.1. Primary Methods for In Vitro and Ex Vivo Stiffness Quantification

In biological systems, stiffness can be assessed through a range of approaches, including molecular and cellular correlates of stiffness as well as direct mechanical characterization. In the context of the ECM, molecular and cellular stiffness correlates are typically examined through analysis of transcriptional changes, protein abundance or immunostainings. Transcriptional changes in ECM-related genes can be assessed by RNA sequencing or qPCR to identify differentially expressed genes and their quantitative change in expression, respectively. Altered expression of genes that are related to sensing ECM stiffness or mechanotransduction pathways can suggest ECM stiffness increases or decreases as these genes respond dynamically to mechanical cues [57,58]. Changes in ECM-related protein abundance, assessed f.i. by Western blotting, can indicate cellular adaptations to mechanical changes in the TME [24]. Immunostainings using ECM component-specific antibodies provide insights into the localization and abundance of proteins that contribute to ECM stiffness. These techniques are applied to in vitro cell culture models, ex vivo patient tissue slices or tissues obtained from in vivo animal studies [46,59,60,61].

While molecular changes can serve as indirect indicators of ECM stiffness, direct mechanical characterization provides more explicit and quantitative information about the tissue’s mechanical properties. Techniques to measure local stiffness include, amongst other things, indentation and atomic force microscopy (AFM). AFM is used to quantify tissue stiffness by performing nanometer-scale indentations on tumor tissue and measuring the force required to deform tissue, providing spatially resolved stiffness maps and allowing mechanical characterization of heterogeneous tumor regions [62]. Often, patient tissue samples are probed in order to identify the stiffness of tumor regions as well as healthy brain sections [63]. AFM-based force-distance measurements provide high-resolution and localized stiffness measurements of single cells, organoids or tissue slices. Further, AFM has proven valuable in multiple cancer-related research fields, including characterization of structural and morphological changes in cells, investigation of the tumor microenvironment and biomarker detection [64]. Combining AFM with complementary nanoscale stiffness measurement techniques, such as optical tweezers, can improve mechanical phenotyping. This integrative approach allows for probing cancer cells and tissues across different force scales and spatial contexts, with AFM mapping local stiffness and topography and optical tweezers facilitating single-cell manipulation and measurements at small force [65,66,67]. Additionally, integrating AFM and microfluidic devices that replicate features of GB’s complex TME, such as shear stress, can further strengthen mechanical studies by enabling direct assessment of how shear forces and microenvironmental constraints modulate cell stiffness and adhesion [68,69]. Indentation measurements use larger probes and higher forces, providing an averaged stiffness readout of larger, but still localized, tissue volumes, thus bridging the scale between AFM and bulk stiffness measurements [63,70]. Bulk (tissue) stiffness can be measured using rheometry or low load compression testing (LLCT). LLCT determines stiffness by compressing a sample with controlled forces, measuring the resulting deformation and thus inferring the bulk stiffness [71]. Rheometers apply shear stress to the entire sample volume and measure deformation and resistance to the applied stress, from which the stiffness can be calculated [72]. This macroscopic technique is frequently used to assess the stiffness and viscoelasticity of hydrogels or whole tissue slices.

### 3.2. Hydrogel-Based In Vitro Models

Hydrogels are usually composed of either natural polymers such as HA, collagen and Matrigel or synthetic polymers like polyethylene glycol (PEG). While natural polymers often retain native biochemical cues for GB cells, synthetic polymers offer the opportunity to precisely control mechanical properties independent of biochemistry and bioactivity [73,74]. Stiffness of the hydrogels can be tuned by polymer and water content, and adapted crosslinking density, mimicking soft (40 Pa) to stiff brain ECM (up to 26 kPa) [49].

Hydrogels are used to test the effect of mechanical changes on cellular processes, providing a variable, biomimetic platform to replicate the brain tumor environment and explore the effect of mechanical changes such as stiffness on GB cell morphology, migration and proliferation. Importantly, soft hydrogels can be used as cell culture substrates to mitigate the effect of rigid plastic culture surfaces [75]. When exposed to stiffnesses up to 26 kPa in 3D hydrogels with varying PEG content, Wang and colleagues [49] found U87 cells transforming in a spindle-like morphology with protrusions rich in actin, characteristic of a migratory/invasive phenotype. Indeed, when testing the migratory capacity of GB cells in hydrogels with varying stiffnesses, decreasing matrix stiffness led to reduced migration of GB cells [46,76,77]. In line with these findings, migration speed increased when cells were exposed to higher matrix stiffness [46,78]. Together, these studies support the hypothesis that higher (matrix) stiffness facilitates GB cell migration and invasiveness.

Cell proliferation is another cellular process that can be modulated by the mechanical properties of the surrounding matrix. However, the relationship between stiffness and proliferation is not straightforward. In 2D cultures, several studies report increased proliferation on stiffer substrates and reduced proliferation on softer ones [77,79,80]. In contrast, opposite trends have been observed in 3D systems for example, decreased hydrogel stiffness promoted higher proliferation in U87 cells embedded in 3D hydrogels [49]. These discrepancies likely reflect fundamental differences between 2D and 3D growth environments: in 3D, cells are fully surrounded by the matrix, encounter mechanical confinement and stress transmission that more closely mimic in vivo conditions, and may also experience reduced nutrient and oxygen diffusion in denser, stiffer hydrogels. Together, these factors can alter how GB cells sense and respond to stiffness.

Hydrogels can be used to investigate the mechanical properties of single GB cells [81]. Further, mechanical characterization revealed clear differences between GB cells and other cell types, including reduced cytoskeletal stiffness and smaller focal adhesions in GB cells [82]. Among GB cells, nanomechanical differences between T98G and U87 cells have also been linked to distinct migration patterns [83]. In addition, IDH-mutant GB cells show higher stiffness, suggesting that mechanical profiling may help identify GB subpopulations and support diagnosis [84]. Overall, these findings highlight nanomechanics as a useful marker of GB heterogeneity and behavior at a single cell level. While organoids offer a more sophisticated model to study, for instance oxygen or nutrient gradients, AFM measurements on organoids are limited. Emerging studies provide protocols showing that these types of measurements are possible, simultaneously proposing standardized experimental approaches [85].

Taken together, the effect of mechanical ECM alterations, specifically stiffening, on GB cell behavior can be studied using 2D and 3D hydrogel models, showing that stiffer substrates result in increases in cellular migration and changes in proliferation. Nonetheless, hydrogels provide a simplified TME that lacks heterogeneity and where cell-ECM interactions depend on hydrogel composition.

### 3.3. Ex Vivo Tissue Stiffness Measurements

The studies discussed above made use of in vitro 2D and 3D GB models to mimic the biophysical conditions of tumors, particularly matrix stiffness. However, the mechanical properties of patient tumors involve multiple cellular components whose complexity is often not reflected in the previously mentioned models. In GB tissue, stiffness involves the presence of various cell types, including neurons, astrocytes and microglia that together produce a complex ECM. For this, ex vivo stiffness measurements of patient-derived tissue or organotypic slices may provide a more complete representation of GB stiffness. In this regard, it needs to be mentioned that resected tissue may show altered stiffness compared to the same tissue in a confined (brain) environment, as, e.g., seen by Svensson et al. [6], where tactile intraoperative perceived stiffness of tumors after craniotomy did not consistently align with pre-operative stiffness assessment, where tumors were confined to the skull. Therefore, in vivo and ex vivo stiffness measurements need to be interpreted with caution.

Several studies using AFM have shown an increase in stiffness in tumor tissue slices from GB patients compared to normal brain. When comparing tumor regions to non-tumor regions, there is a clear increase in stiffness, e.g., from 1.56 kPa to 2.75 kPa in human glioma [86]. Comparing non-tumor tissue with low-grade gliomas and GB tumor regions, stiffness further increased from 1 kPa to 3.8 kPa and 8.6 kPa, respectively [87]. This grade-associated increase in tissue stiffness correlated with poor patient prognosis and enhanced glioma progression [37,87]. Further, non-necrotic GB tumor cores exhibited higher stiffness values and increased abundance of HA and TNC compared with the tumor rim and peritumoral tissue (0.6–1.3 kPa vs. 0.3–0.5 kPa), and elevated HA and TNC expression in the tumor core correlated with decreased patient survival [57,88].

While in vitro and ex vivo stiffness measurements provide mechanistic insights from an often controlled and simplified environment, in vivo stiffness measurements can help capture more complex and physiologically relevant information in native biological environments.

## 4. In Vivo Biomechanics of GB

### 4.1. Primary Methods for In Vivo GB Stiffness Quantification

In vivo brain stiffness measurements are limited to MRE and shear wave elastography (SWE).

SWE is an ultrasound-based method for assessing (bio)mechanical properties of tissues. More specifically, acoustic or mechanical signals generate shear waves that propagate through tissues and cause displacement similar to how water ripples in circles when disturbed. Subsequently, the speed at which these waves travel is used to measure Young’s modulus (elasticity), with stiffer tissue leading to increased shear wave speed. Importantly, SWE is used directly on the dura mater or the brain and therefore requires prior craniotomy, making this an intraoperative-only technique. SWE has been used to assess tumor boundaries or tumor grade based on mechanical properties [89,90,91].

Similar to SWE, MRE uses externally induced low-frequency mechanical vibrations of roughly 50–200 Hz generated by an external actuator, for example, through a specially designed pillow-like device or a head cradle. The shear waves arising from vibrations travel through the brain and lead to microscopic brain tissue displacement, which is captured by a simultaneously performed MRI scan. Brain tissue stiffness is inferred from wave propagation, with higher wave speed indicating stiffer tissues. Using MRE, the shear modulus is frequently provided as a measure of stiffness, representing the tissue’s resistance to shear forces [14,92]. Of note, MRE is used in pre- and post-operative settings and is a non-invasive technique that does not require craniotomy. MRE can be used for surgical planning but its main field of application is research on, e.g., cerebral blood flow in GB tumors [93] or prediction of surgical outcomes in meningiomas [94]. MRE is not included in standard protocols, e.g., GB tumor diagnosis or surgical planning.

Both SWE and MRE have been used to determine GB stiffness in clinical settings, with most studies reporting reduced tumor stiffness compared with surrounding healthy tissue, contrary to the GB ECM stiffness increase reported in the experimental models that were previously discussed. Below, we discuss evidence for GB tumor softness from both in vivo mouse studies and patient-derived data.

### 4.2. GB Softness in Preclinical Mouse Models

GB is commonly modeled in mice using genetic models, even though the focus is shifting to intracranial transplantation of human GB cells, as these recapitulate key features of tumor growth and progression more accurately. MRE-based measurements show that the healthy brain parenchyma of mice exhibited greater stiffness than GB tumors, with representative values of 5.89 and 4.8 kPa, respectively [95,96,97,98]. Moreover, the tissue stiffness around murine tumors seems to increase compared to the rest of the brain [98], indicating a stiffening of the surrounding ECM while tumors themselves soften. An advantage of MRE studies is the opportunity to longitudinally track tumors by repeatedly measuring stiffness following cell transplantation. In such a longitudinal study, tumor stiffness was shown to decrease by 38% from two to four weeks post-transplantation [96]. Further, intratumoral stiffness heterogeneity with necrotic areas and viable tumor cells appearing softer than dense tumor cell areas and blood vessels was observed [96,99]. Together, these investigations revealed that GB tumors progressively soften and exhibit increased elasticity, shifting from a viscous fluid to an elastic solid phenotype over time [96,97,100]. This indicates a development towards a softer but more solid-like tumor, which can be attributed to ECM degradation through MMPs, leading to an increase in glycosaminoglycan production such as HA that binds water and contributes to a more elastic, gel-like phenotype [98,100]. Overall, murine studies provide evidence for a progressive, dynamic decline in tumor stiffness, most likely due to ECM degradation and remodeling.

### 4.3. GB Softness in Human Patients

GB stiffness measurements in patients provide a similar trend as observed in mouse studies. GB tumor tissue is softer (0.64–1.5 kPa) than normal appearing, healthy tissue (1.54–1.81 kPa) [6,93,99,101,102]. Additionally, tumor stiffness decreases as tumor grade increases [72,83]. In a recent study by Svensson and colleagues [6], MRE was used to pre-operatively assess and classify tumor stiffness (or softness), showing a 20% decrease in stiffness in GB tumors compared to normal-appearing white matter. Interestingly, even though MRE tumors seemed to be softer than normal-appearing white matter, intraoperative palpation evaluation by surgeons did not fully align with the pre-operative MRE findings. This suggests that MRE captures different physiological or mechanical properties than those perceived during surgery and that tumor confinement inside the closed cranial cavity may affect stiffness measurements [6]. Furthermore, differential gene expression analysis between stiff and soft biopsies, whose categorization was based on tumor consistency and ease of aspiration during surgery, revealed overexpression of genes involved in ECM reorganization and cellular adhesion in biopsies classified as stiff, which provides evidence of involvement of ECM in tumor stiffness.

Overall, GB tumors have significantly reduced stiffness compared with normal brain tissue, although the difference is modest. Even though variability exists across studies, none report an increase in GB tumor stiffness relative to healthy brain tissue, and the substantial stiffening seen in laboratory-based experiments is not reflected in patients. Similarly, GB tumors in mouse studies present the same trend, although absolute stiffness values are higher than in humans. These findings raise the question of why GB tumors are often described as stiffer than normal brain in non-clinical settings, whereas clinical measurements indicate the opposite. In the following section, we will examine potential reasons for this discrepancy and the implications for future research.

## 5. Bridging the Gap: Why Models and Clinical Reality Diverge

The findings summarized above indicate that the relationship between GB and (ECM) stiffness is more complex than initially anticipated. While in vitro and ex vivo experiments generally suggest an increase in GB (ECM) stiffness, the majority of in vivo data indicate the opposite. This discrepancy raises important questions about the biological and technical factors responsible for the conflicting results, as well as the consequences for future research directions and potential implications for patient treatment. In this section, we will discuss technical as well as biological reasons for the discrepancy in GB stiffness data.

### 5.1. Technical Factors Contributing to Apparent Stiffness Differences

**Figure 2:**
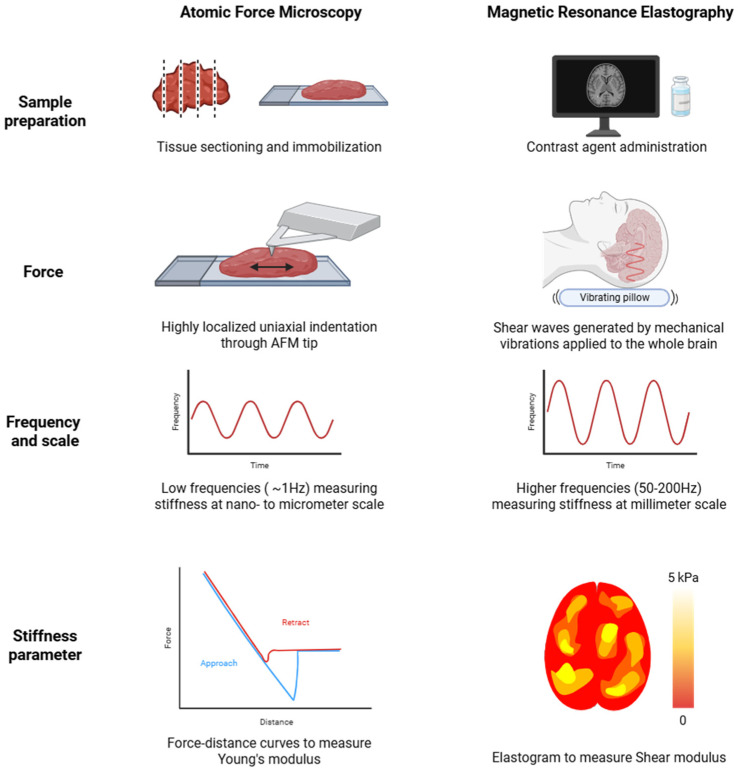
Technical origins of stiffness discrepancy between in vitro/ex vivo and in vivo measurements of GB stiffness. Comparison of technical aspects of Atomic Force Microscopy (AFM), most commonly used on ex vivo GB samples or in vitro GB model systems and Magnetic Resonance Elastography (MRE), commonly used for in vivo GB stiffness assessment.

The most widely used techniques for stiffness measurements in pre-clinical and clinical settings are AFM and MRE, respectively. As outlined earlier, these methods differ fundamentally in how stiffness is detected, which may contribute to the variations in reported stiffness values (Figure 2).

The AFM is a scanning probe microscope which measures stiffness at a nanoscale resolution, while MRE is a tissue-displacement-based, macroscopic tool to assess stiffness. Here, we will discuss the differences between AFM and MRE regarding sample preparation, stiffness parameters, depth of measurements, relation of stiffness to ECM, as well as data analysis and explore how these could influence stiffness values and whether these differences serve as a possible explanation for the apparent stiffness divergence.

#### 5.1.1. Sample Preparation

The AFM requires direct contact with the area of interest and can therefore be performed on freshly excised tissue, retaining its native state apart from the need for mounting and maintaining accurate hydration of the sample during measurements [103]. Frequently, human tumor samples are sectioned to generate sections of suitable size and thickness for AFM measurements [26,37,57] and fixation or cryopreservation may be used when necessary to facilitate handling and sectioning [57,88]. Although snap freezing could theoretically alter ECM and cellular mechanics through ice crystal formation, studies comparing snap-frozen and fresh tissue samples report no significant impact on stiffness measurements in colorectal cancer and lung tissues [104,105]. Nevertheless, sample preparation and handling may still alter their native mechanical state. For example, exposure to room temperature can affect stiffness measurements [106,107,108], similar to sample thickness, which affects the degree of tissue deformation under the AFM indenter and thus stiffness values [109]. These factors indicate a contribution of the sample’s preparation to the measured stiffness values.

Furthermore, samples need to be immobilized to prevent sample movement during measurements. Adhesives such as CellTak, epoxy glues or cyanoacrylate-based glues can be effective [110] but may alter stiffness values through diffusion into the tissue or surrounding medium [111]. For instance, fibrin glue was reported to increase stiffness values [112] and cyanoacrylate caused stiffness artifacts, particularly in already stiff regions [113].

Contrary to AFM, MRE requires minimal preparation. Contrast agents are sometimes administered to enhance MRI contrast for anatomical localization, but major studies report that contrast agents do not alter MRE measurements. Thus, MRE measures tissue stiffness in its native physiological environment.

In summary, AFM sample preparation and ex vivo measurement may alter absolute tissue stiffness and contribute to variability across experiments, whereas MRE requires minimal preparation and captures the physiological state under natural conditions. However, opposite stiffness trends observed between AFM and MRE are more likely due to methodological differences in measurement modalities and scale, rather than preparation effects alone.

#### 5.1.2. Stiffness Measurement Modalities and Principles

AFM and MRE are both used to assess tissue stiffness but use different parameters. AFM typically measures Young’s modulus, reflecting the elastic response to stretching and compression [114]. MRE quantifies stiffness using the shear stiffness parameter, which reflects the tissue’s resistance to shear deformation [14]. AFM and MRE stiffness assessment mainly differ in the mode of tissue deformation. Young’s modulus is obtained by uniaxial stretching or compression of tissue, i.e., when an AFM cantilever is pressing down on a sample, measuring the tissue’s resistance to deformation, with higher resistance indicating higher stiffness [111,114]. The shear modulus measured by MRE also reflects tissue response to deformation but focuses on how force propagates through the tissue. MRE applies mechanical vibrations parallel to the tissue, causing adjacent tissue layers to slide past each other. The stiffness is inferred from the speed of the shear waves, with faster propagation indicating stiffer tissues [14].

Further, the analysis of stiffness values differs between AFM and MRE measurements. AFM calculates the Young’s modulus, and thus stiffness, by fitting force-indentation curves to mechanical models that describe the tip-sample interaction. These models presume ideal tip geometry and a homogenous, elastic tissue, which oversimplifies the complex, viscoelastic nature of biological tissues and can lead to overestimated stiffness values [115,116,117]. It is important to note that for AFM measurements, probe selection and tip morphology need to be considered, as these can influence measured values (discussed elsewhere [85,118,119]).

In contrast, the complex shear modulus obtained during MRE measurements includes both elastic and viscous components. However, similar to AFM, the inversion algorithms used in MRE do not account for the brain’s heterogeneous and anisotropic characteristics. Further, shear wave propagation can be disturbed by cerebrospinal fluid, potentially causing local underestimations of stiffness near fluid-solid interfaces such as the skull [120]. Standard noise-reduction and wave-enhancement filters used in MRE can additionally lead to decreased values by averaging values over larger areas [121]. As a result, AFM generally yields higher apparent stiffness values due to geometric confinement and simplified indentation modeling, whereas MRE often reports lower values due to spatial averaging and increased signal attenuation that occurs at higher vibration frequencies.

Together, AFM and MRE obtain different stiffness parameters that might not be comparable, due to the inherent differences in tissue deformation as well as data analysis.

#### 5.1.3. Force and Scale of Stiffness Measurements

In addition to using different stiffness parameters, MRE and AFM also differ fundamentally in how force is applied to generate stiffness measurements. The AFM operates at low frequencies (e.g., 1 Hz) [88], leading to localized deformations on the nano- to micrometer scale. This allows for high-resolution measurements providing information about local tumor heterogeneity and stiffness differences between tumor core vs. rim [88]. In contrast, MRE applies force at higher frequencies ranging from 50 to 200 Hz, causing broader tissue displacement and enabling stiffness measurements over larger areas. This gives information about tumor stiffness at the millimeter scale and provides greater depth penetration than AFM, although with reduced spatial resolution. MRE measurements detect intratumoral stiffness heterogeneities and can help identify necrotic cores [6,93,94,122].

The differences in force application and measurement area could affect the resulting stiffness values. AFM uses a localized, compressive force through tip indentation, probing a geometrically confined region. Because the sample is attached to a rigid substrate, lateral expansion is restricted, which can result in overestimation of stiffness values [123]. On the other hand, MRE experiences minimal geometrical confinement, as shear waves propagate freely throughout the tissue.

Moreover, with regard to the penetration depth, the AFM mainly probes ECM stiffness at the tissue surface, whereas MRE measures whole tumor stiffness, i.e., of cells plus ECM, which requires additional techniques such as differential gene expression analysis to be related to ECM stiffness [6].

Taken together, AFM stiffness values might be skewed towards increased values due to localized force application and geometrical confinement, factors negligible for MRE measurements. This further highlights the challenges associated with directly comparing these two techniques.

While AFM and MRE do not provide directly comparable stiffness values, they nevertheless both reflect tissue mechanical properties. Although sample preparation, measurement scale and differences in mechanical parameters all influence the stiffness values reported by different techniques, these factors alone do not fully account for the large discrepancies reported in the literature. This suggests that additional biological factors contribute to the divergence between in vivo and in vitro/ex vivo measurements.

### 5.2. Biological Factors Underlying Softness in Human GB

**Figure 3:**
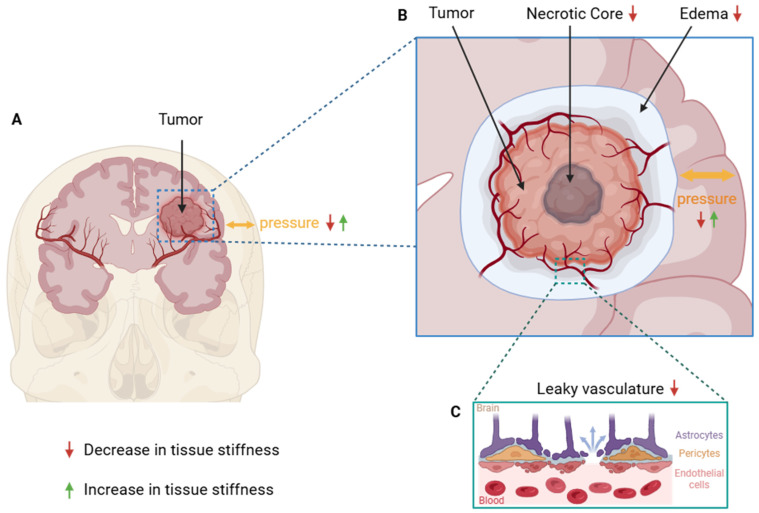
Biological factors influencing mechanical properties of brain tumors. Red arrows indicate a decrease in tissue stiffness, while green arrows indicate an increase. (**A**) shows that with tumor growth, intracranial pressure can rise due to the brain’s confinement by the skull, which can affect tissue stiffness. (**B**) shows a schematic of a tumor, whose necrotic core and surrounding edema decrease tissue stiffness. (**C**) shows leaky tumor vasculature which can contribute to a decrease in tumor stiffness due to a local increase in edema and interstitial pressure.

While technical reasons can partly account for the discrepancy in stiffness measurements, biological factors likely contribute as well. These features may underlie the clinically observed softness in GB tumors—features often absent or underrepresented in pre-clinical models (Figure 3). Amongst these factors are necrosis, a leaky vasculature, edema and intracranial pressure, as well as tumor heterogeneity. The following section examines these factors in detail and how they contribute to the characteristic softness of GB tumors.

#### 5.2.1. Necrosis

Necrosis in tumors is largely driven by nutrient and oxygen depletion caused by inadequate vascularization. Necrotic cell death can arise from glutamate accumulation in peritumoral fluid due to reduced uptake by GB cells and subsequent excitotoxicity [124,125]. The necrotic process results in loss of cell and ECM integrity, due to ECM-degrading enzymes such as MMPs being more abundantly expressed in hypoxic conditions and thus contributes to mechanical alterations [126]. In vitro findings using AFM show that necrotic tumor cores are softer than surrounding GB tissue [16,26]. Similarly, necrotic tumor areas in mice as well as in humans assessed by MRE were shown to have decreased stiffness compared to surrounding tumor tissue [6,96]. Together, this suggests that necrotic regions in GB tumors are softer than surrounding tumor tissue across both clinical and pre-clinical settings and independent of measurement techniques. Therefore, although necrosis contributes to overall tumor softness, it does not account for the discrepancies observed between pre-clinical and clinical settings.

#### 5.2.2. Leaky Vasculature and Edema

The integrity of the blood–brain barrier and brain vasculature is disrupted in GB. Damage to the blood–brain barrier causes reshaping through released factors such as vascular endothelial growth factor (VEGF) or MMPs, creating a blood-tumor barrier [127,128]. This barrier is more permeable, allowing for fluids to enter the extracellular space around tumor cells. Leaky vasculature can further contribute to peritumoral edema, an excessive buildup of extracellular fluid around the tumor [10,127]. Edema is especially prevalent in recurring GB tumors but can also be found in newly diagnosed GB and has been associated with worse survival [129,130,131,132]. Tissue stiffness decreases when extracellular fluid accumulates, because the solid components of the tumor get diluted [133]. Moreover, computational modeling has shown that tissue deformation through fluid pressure decreased tumor stiffness [134]. Although some orthotopic and patient-derived glioblastoma models can reproduce vascular permeability and edema, these features are often absent or incompletely represented in in vitro pre-clinical models. Consequently, the absence of these fluid-associated features in experimental systems may help to explain the discrepancies that are observed between GB stiffness measurements in vitro/ex vivo and in vivo.

#### 5.2.3. Intracranial Pressure and Confinement

Leaky vasculature and edema, as well as the growing tumor itself, can contribute to increases in intracranial pressure (ICP) due to fluid build-up, insufficient drainage from the skull, and tissue displacement [127,135]. Although elevated ICP would intuitively be expected to increase brain stiffness through mechanical compression, the relationship appears more complex. Modulation of physiological ICP (2–40 mmHg) in porcine brains did not significantly affect the brain’s shear modulus [108], whereas an immediate and significant stiffening of the brain was found when inducing short-term ICP increases in healthy human volunteers. In GB, ICP is thought to rise gradually and nonlinearly over time due to tumor growth and edema accumulation [127,136], making it challenging to determine the direct effect of ICP on brain tissue stiffness, especially because of the technical limits of in vivo longitudinal ICP tracking. A more relevant consideration is whether the presence or absence of physiological ICP could partly explain the stiffness differences observed between in vivo and in vitro measurements. The role of ICP is complex and intertwined with other critical factors, particularly the confinement imposed by the skull. Brain tissue measured in situ (partial confinement to skull, post-mortem) and in vitro (no confinement) is significantly stiffer than tissue measured in vivo [108]. This suggests that removal of the brain from its native, constrained and perfused environment can affect its stiffness. Overall, the direct effect of ICP on brain stiffness remains debated; however, evidence suggests that loss of physiological ICP and skull-derived confinement can increase brain stiffness, hence adding to the discrepancies observed between in vivo and ex vivo stiffness measurements.

#### 5.2.4. Tumor Heterogeneity

In clinical settings, GB tumors exhibit considerable variability in stiffness within and between individual tumors, ranging from a soft gel-like consistency to almost solid [93]. Further, in vitro studies suggest that stiffness can vary between tumor rim and core, attributed to factors such as necrosis, ECM degradation, and heterogeneous cell densities [10,57,88]. Similarly, MRE measurements detected regional intratumoral stiffness differences and spatial stiffness gradients [93,99]. However, in vitro techniques such as AFM typically analyze isolated tissue sections or cell clusters under simplified or artificial culture conditions, which do not capture the mechanical influence such as edema or ICP may therefore incompletely capture intratumoral heterogeneity. On the contrary, MRE measurements offer a more global stiffness assessment which can better capture mechanically heterogeneous areas. Tumor heterogeneity complicates the interpretation of biomechanical data, particularly when comparing preclinical in vitro models to clinical in vivo measurements and can thus cause discrepancies between in vitro and in vivo stiffness measurements.

Despite the fact that GB is a solid tumor, it does not develop a continuous fibrotic capsule as most solid tumors do [137,138]. Since encapsulated tumors retain a rigid, collagen-rich boundary, their ex vivo stiffness (e.g., measured locally with AFM; [139]) tends to qualitatively more closely match in vivo stiffness (e.g., measured in bulk by MRE; [140]), because both techniques probe the same solid structural components. In contrast, GB lacks such fibrotic capsules, while its in vivo stiffness is strongly influenced by fluid-associated factors such as edema, leaky vasculature and interstitial pressure, features that disappear ex vivo, leading to poor correlation between AFM and MRE measurements.

Together, the discrepancies in GB stiffness measurements between pre-clinical and clinical settings likely arise from a combination of biological factors that are not properly captured in experimental models. These include necrosis, vascular permeability leading to edema, elevated intracranial pressure due to skull confinement, and the inherent heterogeneity of the tumor microenvironment. By altering fluid balance, pressure, and structural integrity, these features can locally soften or stiffen tissue. Their absence or rather underrepresentation in pre-clinical models likely contributes to the stiffness differences observed relative to clinical measurements.

## 6. Conclusions and Future Directions: Toward Mechanically Faithful GB Models Through the Integration of Biomechanics into GB Research

**Figure 4:**
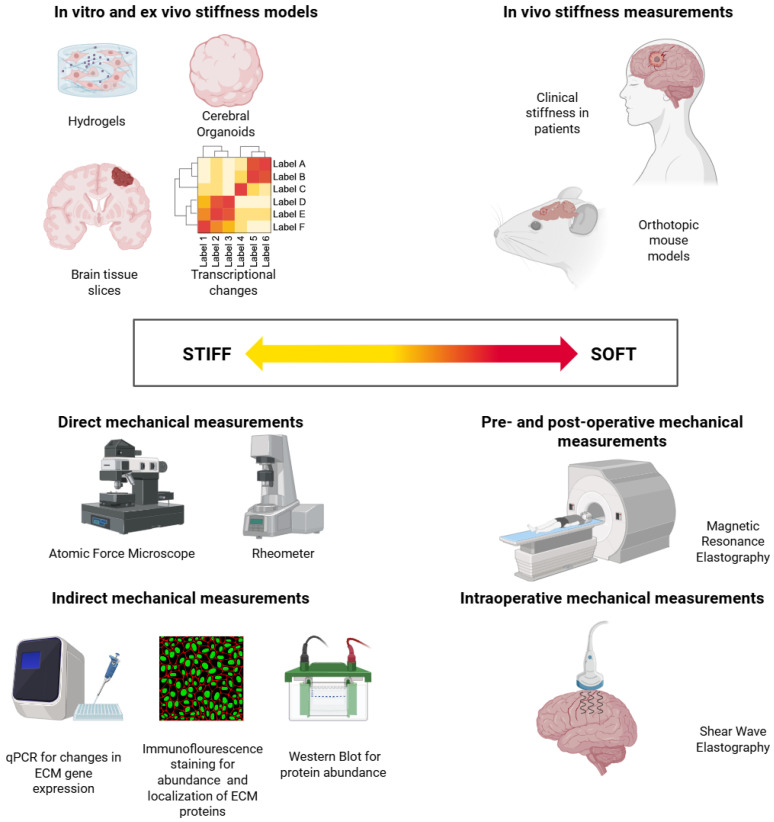
Overview of stiffness measurements in pre-clinical and clinical settings reporting opposing stiffness values of glioblastoma tumors.

GB remains incurable due to highly aggressive biological behavior and therapy resistance. A major factor influencing GB invasive growth and tumor progression is mechanical alteration in the tumor and TME through ECM stiffening. However, pre-clinical and clinical measurements report opposing stiffness values, with pre-clinical assessment showing higher tumor stiffness than clinical assessment. This discrepancy arises from both technical and biological factors, summarized in Figure 4. While no single factor can be identified as the primary driver of stiffness discrepancy, it is most likely the result of the interplay of technical and biological factors together.

Resolving the discrepancy between pre-clinical and clinical GB stiffness is pivotal, as misinterpretations about the effect of tissue stiffness on tumor cell invasion and proliferation can hinder accurate representation of mechanical properties and their translation to clinical reality. Thus, it is crucial to develop and use accurate pre-clinical models and measurement techniques of GB stiffness.

One of the main remaining challenges is the methodical differences between preclinical stiffness measurements and clinical assessments, typically relying on AFM and MRE, respectively. These techniques are inherently different in scale, biological context and sample preparation, thereby limiting direct comparison. Pre-clinical studies typically measure stiffness ex vivo using AFM, which tends to overestimate stiffness due to geometric confinement and model assumptions. In contrast, clinical measurements rely on MRE, which can underestimate stiffness because of spatial averaging. Differences in sample preparation and the spatial scale further contribute to these opposing values. Biological factors also play a major role, as some key features present in patients, such as necrosis, edema, intracranial pressure and tumor heterogeneity, are often absent or underrepresented in pre-clinical models.

In vivo-ex vivo correlation animal studies that apply MRE, followed by brain extraction, tissue preparation and subsequent AFM measurements could help calibrate and better resolve the observed discrepancies. Further, standardization in reporting stiffness values (e.g., standardization of AFM sample preparation) and methodical information could help improve (cross-) method comparability. Future work aimed at resolving technical differences by integrating pre-clinical and clinical stiffness assessment in a longitudinal manner may help clarify and partly resolve these differences.

In addition, GB stiffness likely changes over time due to tumor growth, necrosis, edema and ECM remodeling. Current models are often static and overlook these temporal stiffness dynamics and may therefore miss critical transition points that can inform about underlying biological processes. Longitudinal stiffness measurements in living organisms could help clarify how tumor mechanics develop over time and how these changes relate to tumor progression and invasion. Repeated MRE in mice bearing GB tumors, combined with ex vivo mechanical mapping could help track temporal changes in tumor stiffness. Additionally, repeated, non-invasive, label-free measurements using Brillouin microscopy in transparent zebrafish larvae [141,142], which infer mechanical properties such as viscoelasticity from the frequency shift in scattered light, may provide complementary insight into tumor stiffness evolution over time.

Next to improving clinical stiffness detection, preclinical models could also benefit from adaptations. Hydrogels provide a simplified environment with oftentimes defined stiffness which can be used to study mechanical influences on GB cell behavior. Ideally, preclinical models should reproduce the spatial and mechanical heterogeneity that is observed in clinical GB. While incorporation of GB stem-like cells offers higher physiological relevance regarding tumor growth and invasiveness, simple changes to softer substrates (e.g., Matrigel, fibronectin) in 2D and 3D cell cultures would represent a more physiological, soft environment for the GB cells to grow in. Additionally, assemblies between GB spheroids and cerebral organoids or genetically engineered cerebral organoids developing GB [143,144,145] provide an intermediate model between preclinical and clinical GB research and offer an adaptable platform to study GB stiffness. With cerebral organoids, processes such as spatial stiffness heterogeneity (soft, necrotic core, stiff rim) can be modeled [146]. Further, biological complexity can be created through co-culture with immune cells or vasculature [147], and biological features such as increased pressure or necrosis can be simulated through physical encapsulation/compression through hydrogels or fibroblast co-culture [148,149] or introduction of oxygen gradients [150], respectively. Even though standardized experimental approaches for measuring the stiffness of organoids use AFM, their implementation is still ongoing [85].

Lastly, the implications of stiffness discrepancy extend beyond biophysical modeling and have direct consequences for therapeutic development and clinical translation. Stiffness affects therapy response, including radiotherapy and drug penetration, through mechanical barriers, increased intracranial pressure and abnormal vasculature [151,152]. Thus, accurate modeling of stiffness is crucial for testing drugs and delivery systems as well as revealing potential new therapeutic targets. As GB stiffness may influence drug delivery and activate mechanotransduction pathways that promote cell migration and invasiveness, pharmacological targeting of mechanotransduction could help reduce invasiveness and potentially limit tumor recurrence.

Reproduction of physiologically relevant GB properties in vitro remains challenging; hence, robust in vivo systems are required. Ideally, such models would offer higher throughput than conventional mouse models, particularly regarding efficient drug screenings as the median survival rate of GB patients under standard treatment protocol has not improved during the past 20 years [153]. In this context, the zebrafish offers a promising alternative to traditional mouse models. Larval orthoptic xenografts provide a high-throughput platform with strong potential for drug testing (see, e.g., [154]). Moreover, with cranial development starting as early as 10–14 days post-fertilization [155], confinement and pressure on GB tumors could be partially recapitulated.

Addressing the discrepancy between preclinical and clinical GB stiffness by curating accurate model systems to investigate disease mechanisms and to advance GB treatment is essential, as mechanical changes in GB influence tumor behavior, therapeutic response and ultimately patient outcomes.

## Data Availability

No new data were created or analyzed in this study.

## Abbreviations

The following abbreviations are used in this manuscript:

AFM	Atomic Force Microscope
ECM	Extracellular Matrix
FN1	Fibronectin1
GB	Glioblastoma
HA	Hyaluronan
ICP	Intracranial Pressure
LLCT	Low Load Compression Testing
MMP	Matrix Metalloproteinase
MRE	Magnetic Resonance Elastography
MRI	Magnetic Resonance Imaging
PEG	Polyethylene Glycol
SWE	Shear wave Elastography
TME	Tumor Microenvironment
TNC	Tenascin C
VEGF	Vascular Endothelial Growth Factor
